# Discovery of Antifungal and Biofilm Preventative Compounds from Mycelial Cultures of a Unique *North American Hericium* sp. Fungus

**DOI:** 10.3390/molecules25040963

**Published:** 2020-02-20

**Authors:** Xun Song, François Gaascht, Claudia Schmidt-Dannert, Christine E. Salomon

**Affiliations:** 1 Center for Drug Design, University of Minnesota, Minneapolis, MN 55455, USA; xsong@umn.edu; 2 Department of Biochemistry, Molecular Biology and Biophysics, University of Minnesota, Minneapolis, MN 55455, USA; fgaascht@umn.edu (F.G.); schmi232@umn.edu (C.S.-D.)

**Keywords:** edible mushroom, *Hericium americanum*, *erinaceum*, alpestre, abeitis, antifungal, chlorinated orcinol derivatives, antibiofilm, basidiomycota

## Abstract

Edible mushrooms are an important source of nutraceuticals and for the discovery of bioactive metabolites as pharmaceuticals. In this work, the OSMAC (One Strain, Many Active Compounds) approach was used to isolate two new compounds (**1** and **2**) along with seven known compounds (**3**–**9**) from a mycelial culture of a unique North American edible mushroom *Hericium* sp. The fruiting body was collected in Marine on St. Croix, Minnesota (USA), and mycelial cultures were grown on four different solid and liquid media. Extracts from the mycelial cultures were screened for antimicrobial activity and only the extract from the Cheerios substrate culture exhibited antifungal activity. Bioassay guided fractionation and HPLC analysis were used to isolate nine pure compounds and the structures of the known compounds were established by analysis of the NMR and mass spectrometry data and comparison to published reports. Compound **1** is a new erinacerin alkaloid and **2** is an aldehyde derivative of 4-hydroxy chroman. Four chlorinated orcinol derivatives (**3**–**6**), a pyran (**7**), erinaceolactone (**8**), and erinacine (**9**) were identified. Compound **4** showed antifungal activity against *C. albicans* and *C. neoformans* (MIC = 31.3–62.5 μg/mL, respectively). Compound **4** also inhibited biofilm formation of *C. albicans* and *C. neoformans* at 7.8 μg/mL. These results suggest that mycelial cultures of edible fungi may provide useful, bioactive compounds.

## 1. Introduction

The fruiting bodies (mushrooms) of Basidiomycota have been used as tea, health enhancing foods, and medicines for thousands of years as an integral component of traditional folk medicine cultures globally, including Traditional Chinese Medicine (TCM) [1,2,3,4]. Although production of important clinical antibiotics such as penicillin, cephalosporin, and griseofulvin by filamentous Ascomycota fungi is well known [5], a number of potent, biologically active secondary metabolites have been isolated from Basidiomycota and these compounds may contribute to their medicinal usage [6]. For example, psilocybin and psilocin from a wide range of mushroom species are being clinically tested for treatment-resistant depression and anxiety disorders [7,8]. Due to their chemical diversity and historical success as both medicines and sources of bioactive compounds, mushrooms remain an attractive resource for natural products drug discovery.

The fruiting bodies and substrate mycelia of mushroom forming fungi are spatially and ecologically separate, with different environmental pressures and defensive strategies [9]. It is likely that these morphologically distinct forms differentially upregulate secondary metabolite pathways [10]. Some mushrooms may utilize the production of secondary metabolites for chemical defense from animal and insect grazers, plant or fungal competitors, or for protection from bacterial, fungal and viral infection [11]. The mycelial forms of basidiomycete fungi are generally found within the surface substrate (soil, wood, decaying plant material), and are subject to different competitors and grazers including other fungi, bacteria, and insects [9,12].

Historically, the fruiting bodies of mushrooms are typically cultivated or wild collected and used for medicinal purposes or food. For some valuable species, sporadic growth under specific ecological and weather conditions, rare availability, or lack of cultivation systems make fruiting bodies unreliable and expensive. Many of these strains can be readily grown as mycelial cultures and manipulated and investigated under laboratory conditions as an expansive resource for the discovery and production of compounds of interest. However, under such in vitro cultivation conditions, expression of bioactive secondary metabolites is often different than in the fruiting bodies, reduced, or in many cases even totally silenced [13,14]. The OSMAC (One Strain, Many Active Compounds) strategy aims to trigger the production of secondary metabolites by controlled-stimuli including cultivation on different media, variation of growth temperature or media pH, or the use of chemical or biological agents as elicitors [15]. Multiple cultivations of the same fungal strain under different conditions can therefore lead to the production of different compounds, including new antimicrobial lead compounds.

*Hericium* species such as *H. erinaceus* and *H. americanum* are Basidiomycota fungi found on decaying hardwood and coniferous trees throughout northern United States and Canada. These species are considered highly prized edible mushrooms characterized by a single or branched fruiting body, respectively, with long spines [16]. *H. erinaceus* is the most well-known edible and medicinal mushroom of the *Hericium* genus that has been used for centuries as a delicacy in Asian countries [17]. Extracts prepared from the fruiting body of *H. erinaceus* have been reported to display various pharmacological actions, such as neuroprotective, anti-inflammatory, and antioxidant properties [18]. Previous studies on *H. erinaceus* reported that both fruiting bodies and mycelia contain various bioactive constituents such as polysaccharides, lectins, proteins, hericenones, erinacines, erinapyrones, sterols, fatty acids, and esters [19,20,21,22]. Moreover, *H. erinaceus* mycelia enriched with erinacine A was shown to have numerous health benefits, including lifespan-prolonging activity [23] and delay of neuronal cell death in rats with neurodegenerative diseases such as ischemic stroke, Parkinson’s disease, Alzheimer’s disease, and depression [24]. The specimen in our project (*Hericium* sp. WBSP8, Figure S1) shared morphological characteristics with *H. americanum* but is more closely related to other species including *H. abietis* and *H. alpestra* by comparison of ITS sequences, and more work is needed to fully characterize this possible new species. Due to the potential novelty of this specimen and distinction from more well-characterized species, we conducted a detailed chemical and biological activity study. Our objective was to elicit the production of a broad range of secondary metabolites by establishing mycelial cultures on several different liquid and solid substrates (malt, soy, rice, and oat-based Cheerios breakfast cereal). Extracts from mycelial cultures yielded chemical profiles that were different from each other and from that of the original fruiting body from which they were established. Bioassay guided fractionation of extracts for antimicrobial activity resulted in the isolation and structure elucidation of two new compounds (**1** and **2**) and seven known compounds (**3**–**9**). Although the structure of compound **4** was previously reported, we identified new antifungal activities, including prevention of *Cryptococcus neoformans* biofilms at sub-inhibitory concentrations.

## 2. Results

### 2.1. Comparison of Chemical Profiles of Hericium sp. WBSP8 Extracts

**Figure 1:**
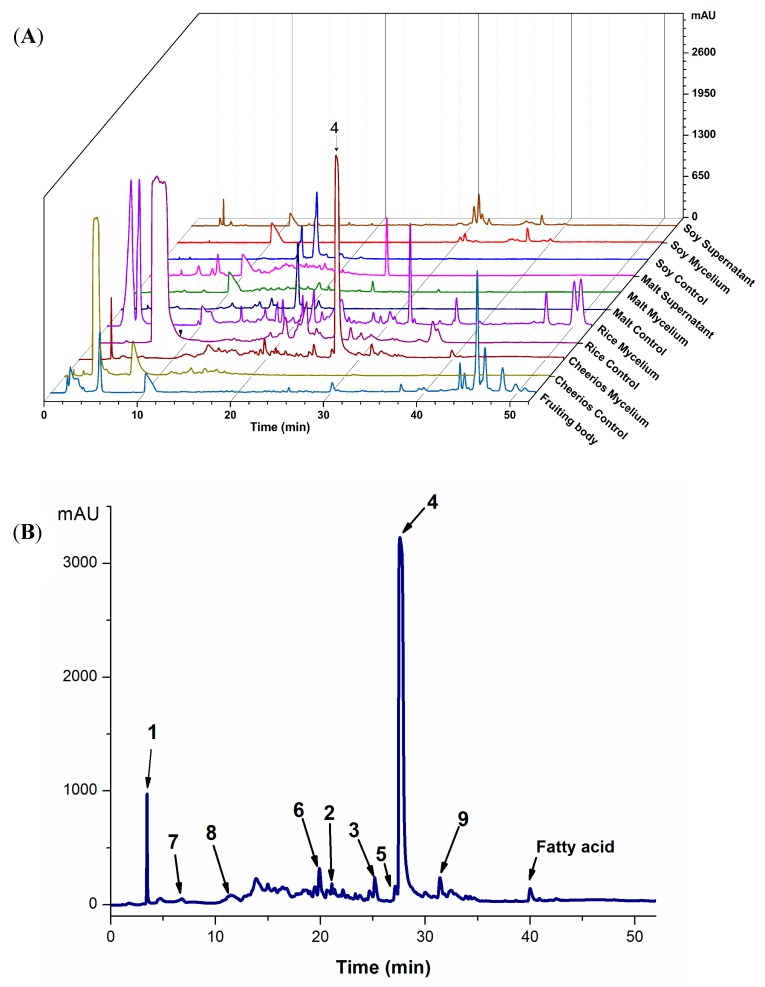
(**A**) Superimposed HPLC-DAD chromatograms at 220 nm of *Hericium* sp. WBSP8 extracts from a fruiting body and different cultures grown in liquid (soy, malt) or solid media (Cheerios, rice). The major peak corresponding to compound **4** (tR = 28 min) is indicated by an arrow. (**B**) HPLC chromatogram of the antifungal Cheerios extract recorded at 220 nm showing peaks corresponding to compounds **1**–**9.**

By applying the OSMAC approach, different cultivation conditions were expected to elicit the production of diverse compounds. The fruiting body, mycelia cultured in solid (Cheerios or rice substrates) and liquid media (malt or soy media), or culture supernatants (soy or malt liquid culture) were extracted and the chemical profiles of the seven extracts were compared by HPLC analysis (Figure 1) and tested for biological activities. Except for shared compound peaks with retention times (tR) between 10-12 min, extracts from mycelial cultures contained several compounds that were not found from the fruiting body of *Hericium* sp. WBSP8. Extracts from the fruiting body primarily yielded peaks (tR = 44–50 min) corresponding to relatively nonpolar compounds. Liquid cultures resulted in similar chromatographic profiles for both mycelial and supernatant extracts.

Cultures grown on rice or Cheerios also produced a range of compounds with medium polarity (tR = 20–30 min), including a prominent peak at tR = 28 min present only in the Cheerios extract.

### 2.2. Analysis of Antimicrobial Activities of Hericium sp. WBSP8 Extracts

**Figure 2:**
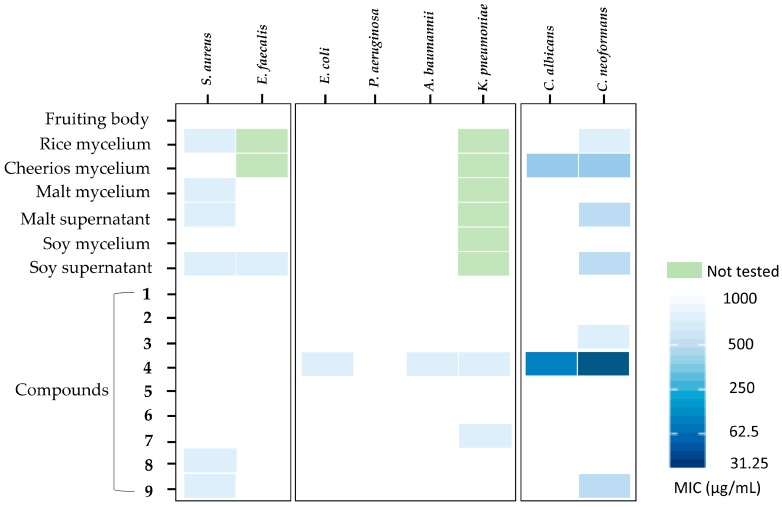
Antimicrobial activity of *Hericium* sp. WBSP8 extracts and compounds **1**–**9** against a panel of eight human microbial pathogens shown as minimal inhibitory concentrations (MIC, μg/mL).

The seven extracts in Figure 1A were screened for activity against a panel of six bacterial and two fungal human pathogenic strains using the microbroth dilution method (Figure 2). Extracts were tested at a wide range of concentrations up to 1 mg/mL because we are aware that many people consume dried mycelial and mycelial extract formulations from edible mushrooms that might contain comparable quantities of active metabolites [25]. Only the crude extract from the Cheerios culture showed activity against both yeast pathogens *Candida albicans* and *Cryptococcus neoformans* with an MIC of less than 250 µg/mL. Extracts from soy and malt culture supernatants showed activity against *C. neoformans* with MICs comparable to the Cheerios extract, while extracts from rice cultures were only weakly active (MIC > 500 µg/mL). Several extracts (rice, malt mycelia and supernatant, and soy supernatant) exhibited weak antibacterial activity (MIC > 500 µg/mL) against *S. aureus* and the soy culture supernatant extract was also active against *E. faecalis* (MIC > 500 µg/mL). The fruiting body extract was inactive against all of the strains tested.

### 2.3. Bioassay-Guided Isolation of Secondary Metabolites from Hericium sp. WBSP8 Grown on Cheerios

**Figure 3:**
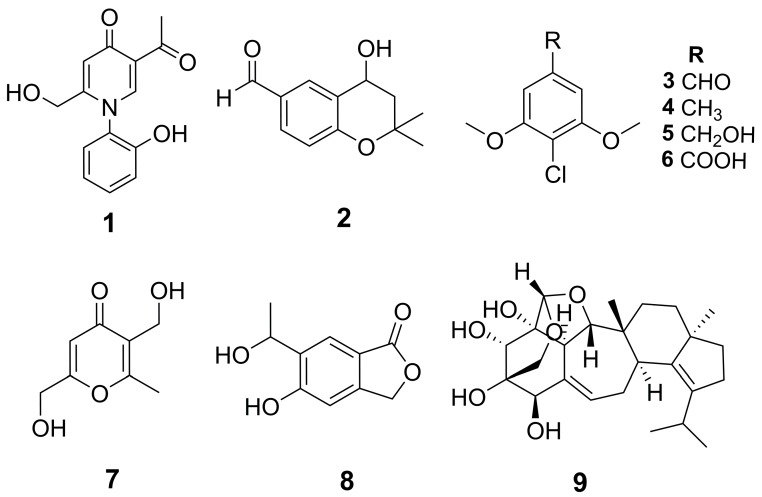
Structures of isolated compounds **1**–**9** elucidated by NMR (Table 1 and Table 2).

We used bioassay-guided fractionation on the most active extract from the Cheerios culture to isolate, identify, and characterize the inhibitory compounds. In addition, the HPLC profile of this extract displayed a prominent, unique peak at tR = 28 min, which was likely related to its antifungal activity. The Cheerios culture was extracted successively with ethyl acetate (EtOAc), acetone, and methanol (MeOH) (3 × 400 mL each solvent). The extracts were combined, dried, and subject to a modified Kupchan partition scheme. The active EtOAc fraction was separated using reverse-phase and Sephadex LH-20 column chromatography, followed by semi-preparative RP C_18_-HPLC to provide metabolites **1**−**9** (Figure 3 and Figure S2).

### 2.4. Structural Identification of Isolated Compounds

**Figure 4:**
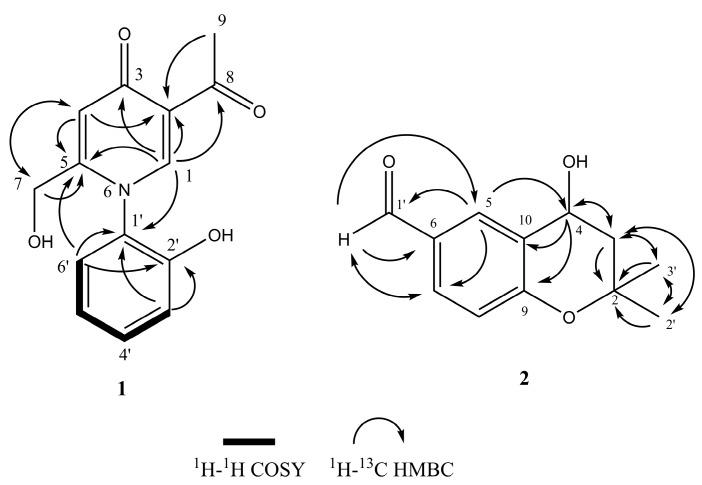
Selected HMBC and ^1^H-^1^H COSY correlations of compounds **1** and **2**.

Compound **1** was obtained as light yellow amorphous solid. The molecular formula of **1** was deduced as C_14_H_13_NO_4_ (nine degrees of unsaturation) on the basis of its HRAPCI-MS molecular ion at *m*/*z* [M − H]^−^ 258.0776 (calc. 258.0772). The 1D and 2D NMR spectra of compound **1** indicated one methyl (C-9), one oxygenated methylene (C-7), six aromatic methines (C-1, C-4, C-3′–C-6′), and two carbonyl moieties (C-3, C-8) (Table 1). Analysis of the HMBC correlations (Figure 4) and comparison of the NMR data to published data for erinacerins N, O, and P confirmed the presence of a 2-acetyl-5-(hydroxymethyl) pyridin-4(1*H*)-one subunit [26]. The remaining signals were attributed to a di-substituted aryl ring with four contiguous protons at *δ*_H_ 7.34 (d, *J* = 7.9, 1.5 Hz, 1 H), 7.49–7.41 (m, 1 H), 7.10–7.01 (m, 2 H), and a hydroxy group at C-2′ (*δ*_C_ 154.0). This 2-hydroxyl benzene substituent could only be connected at the C-1′ position via the nitrogen of the pyridinone, and was confirmed by HMBC correlations of H-3′ to C-1′ and C-2′, H-6′ to C-1′, and H-1’ to C-1′. Based on this analysis, the structure of compound **1** was identified as 5-acetyl-2-(hydroxymethyl)-1-(2-hydroxyphenyl)pyridin-4(1*H*)-one and given the trivial name erinacerin V.

Compound **2** was obtained as a white amorphous solid with a molecular formula of C_12_H_14_O_3_. The ^1^H-NMR spectrum of **2** indicated the presence of two methyl groups, one aldehyde proton, and a 1,3,4-trisubstituted benzene ring appearing as an ABX system (Table 2). Analysis of the ^13^C, DEPT, and HMQC NMR data indicated two methyls, one methylene, five methines, three SP_2_ carbons, and one quaternary carbon. The two methyl groups were correlated to each other (H-3′/C-2′, H-2′/C-3′) and to C-2 in the HMBC spectra, indicating their geminal position at C-2 (Figure 4). The HMBC correlations between H-1′/C-6 and H-5/C-1′ established the position of the aldehyde group at C-6. Collectively, these data indicated a dimethyl chromane structure very similar to the plant derived compound 1-[(4*S*)-3,4-dihydro-4-hydroxy-2,2-dimethyl-2*H*-1-benzopyran-6-yl]-ethanone [27], but with an aldehyde at C-6 instead of an ethanone group. In addition, **2** was optically active with a positive specific rotation value of [α]_D_ + 10.0 (c 0.12, MeOH). We compared this data to the published absolute stereochemistry analysis of closely related *R* and *S* C-4 hydroxy chromane enantiomer pairs with a methoxy or ethanone at the C-6 position [28]. In this prior work, these 4*R*/*S* enantiomers were isolated as mixtures from the plant, and could only be separated by chiral HPLC [28]. The optical rotation values of only the 4*S* enantiomers were positive, suggesting the 4*S* configuration for **2** (Table S1). However, as we were not able to analyze compound **2** by chiral HPLC, it is not known if it exists as an enantiomeric mixture, and the absolute stereochemistry of C-4 remains tentative. Thus, **2** was determined to be a novel aldehyde derivative of 4-hydroxy chroman, 4-hydroxy-2,2-dimethyl chromane-6-carbaldehyde.

The compounds **3**–**9** were identified as known compounds 4-chloro-3,5-dimethoxybenzaldehyde (**3**) [29], 2-chloro-1,3-dimethoxy-5-methyl benzene (**4**) [29], (4-chloro-3,5-dimethoxyphenyl)methanol (**5**) [29], 3,6-bis(hydroxyl methyl)-2-methyl-4*H*-pyran-4-one (**6**) [30], 4-chloro-3,5-dimethoxybenzoic acid (**7**) [30], and 5-hydroxy-6-(1-hydroxyethyl)isobenzofuran-1(3*H*)-one (**8**) [31], erinacine *E* (**9**) [32] by comparison of their NMR, MS and optical rotation data with those in the literature (Table S2).

### 2.5. Antibacterial and Antifungal Activity of Isolated Compounds

Compounds **1**–**9** were evaluated for their antimicrobial activities against a panel of eight human pathogens. Compound **4** showed inhibition against *C. albicans* and *C. neoformans*, with MIC values of 62.5 and 31.25 μg/mL, respectively (Figure 2). Compounds **4**, **7**, **8**, and **9** exhibited weak antibacterial activity against several pathogens (Figure 2). Compounds **1**–**3** and **5**–**6** were inactive in all antimicrobial assays.

### 2.6. Antibiofilm Activity of Compounds 1–9

**Figure 5:**
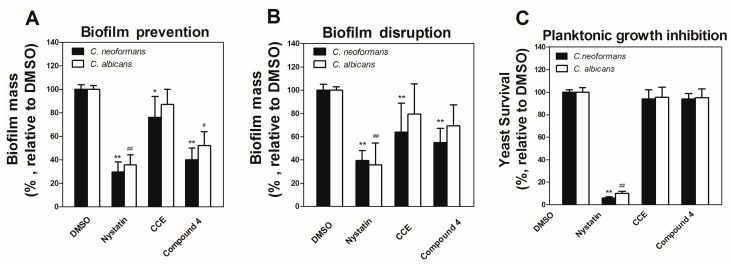
Prevention and disruption of *C. neoformans* and *C. albicans* biofilms by compound **4** and the crude Cheerios culture extract (CCE). (**A**) Biofilm prevention compared to DMSO control at 7.8 μg/mL. (**B**) Disruption of established (24 h) *C. neoformans* and *C. albicans* biofilms at 1 mg/mL. (**C**) Planktonic yeasts growth compared to DMSO control at 7.8 μg/mL. Three independent experiments were conducted. Error bars indicate SEM. (The */** and ^#^/^##^) denotes significant difference between DMSO group and compound-treated group (*p* < 0.01 and *p* < 0.05) for *C. neoformans* (*) and *C. albicans* (#), respectively.

In addition to identifying inhibitors of planktonic microbial pathogens, we are interested in the discovery of compounds that inhibit or disrupt fungal biofilms. Compounds **1**–**9** and the crude extract from the Cheerios culture were tested (0.125–1000 μg/mL) in a biofilm prevention assay with *C. neoformans* and *C. albicans*. Compound **4** reduced biofilm formation by 59.4% and 48.6% for *C. neoformans* and *C. albicans*, respectively, at 7.8 μg/mL (Figure 5A). The crude Cheerios culture extract showed weak activity (23.6% biofilm prevention) at the same concentration (7.8 μg/mL) against *C. neoformans*. In comparison, compound **4** and the crude extract showed no inhibition of planktonic cultures (<10%) at the same concentration (Figure 5C). The same samples were also tested for their ability to disrupt established (24 h) biofilms of *C. neoformans* and *C. albicans* at 1 mg/mL. Compound **4** and the crude extract reduced preformed *C. neoformans* biofilms by 45.0% and 36.1%, while the control compound nystatin reduced biofilm mass by 60.6% at 1 mg/mL (Figure 5B). None of the test samples significantly reduced established *C. albicans* biofilms.

## 3. Discussion

In this work, we compared the chemical profiles and antimicrobial properties of extracts from the fruiting body and in vitro mycelial cultures of *Hericium* sp. WBSP8 cultivated under different conditions. Analysis of extracts by HPLC showed that different cultivation media significantly influenced the production of secondary metabolites. The mycelial culture on Cheerios provided the only antifungal extract and led to the isolation of compound **4** as the major antifungal component (Figure 1), in addition to two novel compounds (**1** and **2**). These results confirm that the secondary metabolome of *Hericium* sp. WBSP8 is distinct when grown under different conditions, consistent with other metabolomic studies of fungi [33,34]. These findings also support the use of Cheerios as a useful cultivation substrate to promote secondary metabolite expression in Basidiomycota fungi, which has been previously shown to enhance the production of novel and diverse compounds in filamentous fungi [35].

Compounds **3**–**9** have been previously isolated from mycelial cultures of *Hericium erinaceus* [36]. Interestingly, we isolated **4** as the primary antifungal component, but it was first isolated from *H. erinaceus* and reported as active against *Bacillus subtilis* and inactive against all fungi tested including *Saccharomyces cerevisiae* IFO0304, *Aspergillus niger* JCM 5697, *Fusarium oxysporum* J1, and *Verticillium dahliae* at 200 μg/disk in diffusion assays [29]. Compound **4** was previously isolated from liquid cultures of *H. erinaceus* with yields of 6.5 mg/L [29], but our cultivation system on Cheerios cereal supplemented with glucose increased this yield five-fold to 38 mg/Kg.

Although compound **4** displayed modest activity against planktonic *C. albicans* and *C. neoformans*, the most significant activity identified was prevention of *C. neoformans* biofilms at sub-inhibitory concentrations. Many pathogenic microbes exist in a biofilm state, which can lead to an increase in virulence, upregulation of toxin production, and increase in drug resistance [37]. *C. neoformans* and *C. albicans* are important pathogens associated with human biofilm infections, and demonstrate significant resistance to clinical antifungals [38,39]. Due to the relatively high toxicity and nonspecificity of most antifungal drugs, fungal biofilm infections are especially challenging to eradicate, and there is a critical need for new biofilm-specific treatments. Compound **4** prevented *C. neoformans* biofilm formation when added to cultures after 6 h of growth, but only disrupted established (24 h) biofilms at very high concentrations (1 mg/mL), suggesting a mechanism related to attachment, cell adhesion, or early signaling [40]. Compound **4** also exhibited no cytotoxicity towards mammalian cells (HaCAT, IC_50_ > 100 μM), in contrast to most clinical antifungal agents. Biofilm preventative compounds similar to **4** could be developed as a safer, less toxic component of coatings for in vivo materials or devices to prevent fungal biofilm growth.

Overall, this study demonstrated that Basidiomycota are a viable source for antifungal compounds and that secondary metabolite production can be elicited in mycelial cultures using similar growth strategies established for other fungi. This work also showed that close taxonomic relatives of well characterized edible mushrooms offer opportunities for new compound discovery.

## 4. Materials and Methods

### 4.1. General Experimental Procedures

Reagents were purchased from Fisher Scientific (Waltham, MA, USA) unless otherwise noted. Optical rotations were measured on a Rudolph Research Analytical Autopol III polarimeter (Hackettstown, NJ, USA). IR spectra were obtained using a JASCO 4100 FT-IR spectrophotometer (Oklahoma City, OK, USA). High resolution mass analyses were performed using an Agilent 6230 TOF LC/MS mass spectrometer (Santa Clara, CA, USA) with a dual ESI and APCI source. Standard 1D and 2D NMR spectra were recorded on a Bruker 400 MHz spectrometer (Billerica, MA, USA). Proton and carbon chemical shifts are reported in ppm and referenced with the ^1^H and ^13^C signals of solvents. TLC separations were performed using Whatman silica gel 60 F_254_ aluminum backed TLC plates. HPLC separations were performed with an Agilent 1200 instrument (Santa Clara, CA, USA) with a PDA detector system.

### 4.2. Mushroom Collection and Identification

A fruiting body of *Hericium* sp. WBSP8 was collected from William O’Brien State Park in October 2016 (Marine on St Croix, MN, USA) and immediately transferred to the laboratory for the establishment of pure mycelial cultures. Briefly, cultures were established on agar plates by collecting several inner pieces (5 × 5 mm) from the fresh fruiting body and transferring them to several Petri dishes prepared with a potato dextrose broth agar medium (HiMedia potato dextrose; HiMedia Laboratories, West Chester, PA, USA; 24 g L^−1^, agar 15 g L^−1^ in distilled H_2_O) under sterile conditions. Petri dishes inoculated with 3–4 explants were then incubated at 25 °C in the dark and observed every two days. Pure fungal cultures were obtained by sub-cultivating outgrowing mycelia. The remaining fruiting body was cut into small pieces (2 × 2 cm) and dried at 40 °C for 72 h. The dried fungal material was stored in a dry and dark environment at room temperature until extraction.

The morphology of the specimen most closely resembled *Hericium americanum* (branching fruiting body with long spines), but molecular analysis suggested that it was distinct from this species. For formal strain identification by internal transcribed spacer (ITS) sequencing, a piece of a well grown mycelia was excised, ground in liquid nitrogen, washed with EtOH, and processed for genomic DNA extraction with buffer based on the protocol established by Saitoh et al. [41]. PCR was performed on genomic DNA with primers ITS1F (5′-CTTGGTCATTTAGAGGAAGTAA-3′) and ITS4 (5′-TCCTCCGCTTATT GATATGC-3′) for amplification of the ITS region [42]. The PCR product was gel purified (Promega Wizard^®®^ SV Gel and PCR Clean-Up System; Promega, Madison, WI, USA) and submitted for Sanger sequencing (University of Minnesota Genomics Center). The resulting ITS sequence was compared by BLASTn analysis to reference sequences in the NCBI Genbank. Analysis of the ITS sequence data show similarity to *H. alpestre* DQ206987 (a European species), *H. abietis* JQ716938 (a species found in the US Pacific Northwest on conifers), and *H. americanum* DQ206987, but is more distant to most other *H. americanum* entries. Due to the complexity of the genus and misidentification of many specimens in the literature and databases, we can only identify our specimen as a unique member of the *Hericium* complex. The fungal culture was incorporated in our Basidiomycota library (University of Minnesota) under the reference 161009-WBSP-0008 and the ITS sequence was deposited in GenBank (accession number: MN243091).

### 4.3. Cultivations

Liquid cultivations were carried out with 125 mL of malt extract media (malt extract (BD Biosciences, San Jose, CA, USA) 30 g L^−1^, soy peptone (Sigma Aldrich, St. Louis, MO, USA) 3 g L^−1^, in distilled H_2_O) or soy medium (Bob’s Red Mill soy flour, Milwaukee, Oregon, USA; 30 g L^−1^ in distilled H_2_O) in a 250 mL Erlenmeyer flask containing a microscope cover glass (22 × 50 mm, Fisherbrand) to dissociate mycelium. For culture inoculation, five pieces of mycelial agar plugs (5 mm diameter) were collected with a Transfertube (Spectrum Laboratories, Inc., Rancho Dominguez, CA, USA), transferred and crushed on the bottom of the flask.

For cultivations with solid substrates, five agar plugs (5 mm diameter) were inoculated into 250 mL-Erlenmeyer flasks containing 32 g of autoclaved Cheerios breakfast cereal (General Mills, Minneapolis, MN, USA) and 100 mL of sterile 0.3% glucose or 32 g of rice and 40 mL of distilled H_2_O.

All cultivations were carried out at 25 °C in the dark for 28 days as static cultures (Rice and Cheerios) and with shaking (150 rpm) for liquid cultures (Malt and Soy).

### 4.4. Pathogens and Culture Conditions

The microbial pathogen strains used for bioassays were methicillin resistant *Staphylococcus aureus* (ATCC 43300), vancomycin resistant *Enterococcus faecalis* (VRE, ATCC51299), *Escherichia coli* (ATCC 25922), *Pseudomonas aeruginosa* (ATCC 27853), *Acinetobacter baumannii* (ATCC 19606), *Klebsiella pneumoniae* subsp. *pneumoniae* (ATCC 13883), *Candida albicans* (ATCC 10231), and *Cryptococcus neoformans* (ATCC 66031). The strains of *S. aureus, E. coli,* and *K. pneumoniae* were grown at 37 °C on Tryptic Soy media (TSA, TSB; BD Biosciences, San Jose, CA, USA). *A. baumannii* was routinely cultured at 37 °C on a nutrient medium (BD Biosciences). Brain heart infusion (BHI; BD Biosciences) was used for the cultivation of VRE at 37 °C. Yeast malt (YM; BD Biosciences) media were used for cultivating *C. albicans* at 30 °C*. C. neoformans* were grown at 30 °C on Sabouraud dextrose media (SDA; BD Biosciences). Strains grown in liquid media were cultivated on an orbital shaker at 200 rpm.

### 4.5. Microbroth Dilution Assay

Compounds and extracts were tested for planktonic microbial growth inhibition using a broth dilution assay [43]. The extracts or compounds were dissolved in DMSO at a stock concentration of 10 mg/mL and kept at 4 °C for the bioassays. Exponentially growing cultures (OD_600_ = 0.03–0.06) of each strain were prepared from overnight cultures. Cultures were diluted 1:10 in broth (*C. neoformans* was used directly without dilutions) and added to a 96-well plate (195 μL/well). Extracts and pure compounds were tested at a final concentration of 250 to 3.9 μg/mL with two-fold dilutions. The samples with inhibition below 50% at 250 μg/mL were tested at a final concentration of 1 mg/mL. Tetracycline (Sigma, St. Louis, MO, USA; 10 µg mL^−1^ in DMSO) was used as positive control against *S. aureus*, *B. subtilis*, *E. coli*, *P. aeruginosa*, *A. baumannii,* and *K. pneumonia.* Penicillin G (Sigma, St. Louis, MO, USA; 10 µg mL^−1^) served as positive control against VRE. Nystatin (Sigma, St. Louis, MO, USA; 10 µg mL^−1^) was used as positive control for *C. albicans* and *C. neoformans*. Plates were read at 600 nm after incubation for 24 h for bacteria and 48 h for fungi. Inhibition was calculated by subtracting the absorbance of the blank wells, dividing by the average value for the DMSO only wells and multiplying by 100.

### 4.6. Tissue Culture Cytotoxicity Assays

Pure compounds were tested against human fibroblast cells (HaCaT) using a standard MTT cell viability assay [44]. HaCaT human fibroblast cells (AddexBio T0020001, San Diego, CA, USA) were maintained in growth media: DMEM (Invitrogen 11965-092, Waltham, MA, USA) supplemented with 10% fetal bovine serum (Invitrogen 16000-044), 1% penicillin/streptomycin (Invitrogen 15140-122), 1% sodium pyruvate (Invitrogen 11360), and 1% Glutamax-1 (Invitrogen 35050-061). 96-well plates were coated with 0.01% collagen overnight and rinsed with PBS prior to seeding with 25 × 104 cells/mL HaCaT cells and allowed to adhere overnight. After 24 h, compounds were added in triplicate at nine, 3x dilutions from 100 μM final concentration in growth media. Plates were incubated for 72 h at 37 °C in a 5% CO_2_/95% air humidified atmosphere after which time the media was removed and MTT was added in a RPMI phenol red free media (Invitrogen 11835-030). The MTT was removed after 3 h, formazan crystals were solubilized with 200 μL of isopropanol and plates were read on an i3 spectrophotometer (Molecular Devices, San Jose, CA, USA) at 570 nm for formazan and 690 nm for background subtraction. EC_50_ values were calculated by fitting the data in the GraphPad Prism software (version 5.04).

### 4.7. Extractions and Compound Isolation

The dried fruiting body was ground to a fine powder, extracted three times with methanol at room temperature overnight, and filtered through Whatman filter paper. The combined filtrates were concentrated, dried with a rotary vacuum evaporator at 40 °C, and stored at −20 °C until use.

The mycelia and substrate from four Cheerios or Rice cultures were combined and successively extracted with ethyl acetate (EtOAc), acetone, and MeOH (3 × 400 mL) with maceration at RT. The organic extracts were combined and evaporated under reduced pressure to afford the dried extracts (6.0 g Cheerios cultures, 18.0 g rice cultures).

Extracts from liquid malt or soy cultures were prepared by combining two 125 mL cultures for each medium and removing the mycelium by filtration through qualitative P8 grade filter paper (Fisher Scientific). The supernatant was extracted three times with an equal volume of EtOAc and then evaporated to dryness. The filtered mycelium was successively extracted with EtOAc, acetone, and MeOH (150 mL) and the combined organic solvents evaporated until dryness.

For HPLC analysis, the samples were prepared by dissolving the extract in MeOH (1 mg/mL), and filtering through nylon 0.2 μm syringe filters (Fisher Scientific). The analysis was conducted with an Agilent Microsorb MV100 RP C_18_ column (250 × 4.6 mm, 5 μm particle size, 25 °C), ultraviolet detection at 220 nm, and 20 μL injections. Samples were analyzed using the following gradient: 0–5 min: 5% acetonitrile (ACN), 5.01–40 min: 5%–100% ACN, 40.01–48 min: 100% ACN, 48.01–50 min: 100%–5% ACN, 50.01–52 min, 5% ACN.

The Cheerios cultures extract (6.0 g) was separated using a modified Kupchan solvent partition scheme with EtOAc/H_2_O, followed by butanol/H_2_O. The ethyl acetate partition was dried (1.2 g), subjected to C_18_ vacuum chromatography (RediSep Gold C_18_ Spherical Silica gel, 300 g, 20–40 μm, Teledyne Technologies, Thousand Oaks, CA, USA), and eluted with a step gradient of MeOH:H_2_O (10%, 30%, 50%, 70%, and 100%) to give five fractions (F1–F5) (Figure S1). Fraction F3 (50% MeOH) inhibited the growth of both *C. neoformans* and *C. albicans* (90.1% and 79.5%, respectively at 250 μg/mL). Therefore, F3 was further separated with MeOH on a Sephadex LH-20 column (Sigma, St. Louis, MO, USA), which led to antifungal fractions F3-3 and F3-4. Subfraction F3-4 (70 mg) was purified by semi-preparative RP-HPLC (0–20 min, 40% ACN, 2 mL min^−1^) to afford **3** (2.0 mg) and **4** (20.0 mg). F1 (50 mg) was separated by semi-preparative RP-HPLC (0–5 min 5% ACN, 2 mL min^−1^, 5–15 min 10% ACN, 2 mL min^−1^) to afford **5** (2.0 mg), **6** (1.1 mg), and **7** (0.9 mg). F2 (50 mg) was further separated by RP HPLC (0–20 min, 25% ACN, 3 mL min^−1^) to afford **1** (2.0 mg), **2** (3.5 mg), and **8** (3.0 mg). Compound **9** (4.2 mg) was isolated from F3-3 by semi-preparative RP-HPLC (0–30 min, 30% ACN, 2 mL min^−1^).

*5-acetyl-2-(hydroxymethyl)-1-(2-hydroxyphenyl)pyridin-4(1H)-one* (erinacerin V) **1**. Light yellow amorphous solid. UV (MeOH): λ_max_ (log ɛ) 315, 245, 230 nm. IR (film): Max 3332, 2940, 2890, 2360, 1680, 1670, 1660, 1034, 1020 cm^−1^. HR-MS (APCI^−^) *m*/*z*: [M − H]^−^ 258.0776 (calcd. for C_14_H_14_NO_4_, 258.0772). For ^1^H and ^13^C NMR spectroscopic data, see Table 1.

*(S)-4-hydroxy-2,2-dimethylchromane-6-carbaldehyde***2**. White amorphous solid. [α]^18^D: + 10 (c 0.12, MeOH). IR (film): Max 3400, 1640, 1440, 1026 cm^−1^. UV (MeOH): λmax (log ɛ) 295, 245, 230 nm. HR-MS (APCI^+^): *m*/*z* 207.1014 [M + H]^+^ (calcd. for C_12_H_15_O_3_, 207.1016). For ^1^H and ^13^C NMR spectroscopic data, see Table 2.

### 4.8. Biofilm Inhibition and Disruption Assays

The fungal biofilm prevention and disruption assays were performed based on published protocols [45] with minor revisions. Biofilms were cultured in flat-bottom, nontissue culture-treated 96-well plates. Overnight cultures of *C. albicans* were grown in YM broth in 15 mL culture tubes at 30 °C on a shaker at 250 rpm for 15 h, cells were diluted to an OD_600_ of 0.5, and then diluted 1:1000 in a RPMI medium (with L-glutamine, without sodium bicarbonate; Gibco, supplemented with 34.5 g/L morpholinepropanesulfonic acid, Sigma and adjusted to pH 7.0 using NaOH). Using the same method, SDB was used as the medium for biofilm formation with *C. neoformans* for all growth steps. Extracts and compounds **1**–**9** were tested at 0.125, 0.5, 2, 7.8, 31.25, 250, and 1 mg/mL in triplicate in both assays. DMSO was used as a negative control for all assays (5 μL).

Prevention of biofilm formation: Diluted cultures of each strain (195 μL) were added to 96-well plates and transferred to a static incubator at 30 °C. Compounds and extracts (5 μL) were added after 6 h, the plates were sealed with parafilm and incubated under static conditions for an additional 42 h at 30 °C.

Biofilm disruption assays: Diluted cultures of *C. albicans* and *C. neoformans* were added to 96-well plates (195 μL) and incubated at 30 °C under static conditions. After 24 h, the planktonic cells and medium were aspirated from the wells and 195 μL of fresh medium was added to the well, followed by 5 μL of test extracts or compounds in DMSO. Plates were sealed and incubated at 30 °C for an additional 24 h.

For both of the above assays, at the end of the final incubation period the planktonic cells and medium were aspirated and wells were washed twice with sterile saline (250 μL). The remaining attached biofilms were fixed with 95% ethanol per well (200 μL), and after 20 min plates were emptied and left to dry. Plates were stained for 15 min with 0.1% crystal violet (100 μL). Excess stain was then removed by rinsing the plate with water. The plates were allowed to dry, and the crystal violet-stained biofilm was solubilized with 200 µL of 30% glacial acetic acid solution per well. The OD of each well was measured at 550 nm with a microplate reader. The percentage of biofilm inhibition was calculated using the following formula:

(1) $$\mathrm{Inhibition}\%=\left(1-\frac{\mathrm{OD}\;\mathrm{sample}-\mathrm{OD}\;\mathrm{blank}}{\mathrm{OD}\;\mathrm{negative}\;\mathrm{control}}\right)\times 100$$

### 4.9. Statistical Analysis

Data are presented as the mean ± standard error (SE). The statistical significance of the data was tested by one-way analysis of variance (ANOVA) using Graphpad Prism 5.0 (Graphpad software Inc., version 5.04, La Jolla, CA, USA). The least significant differences (*P* < 0.05) were calculated to compare differences between the means following determination of significant effects by ANOVA.

## Figures and Tables

**Table 1:** ^1^H, ^13^C NMR, COSY, and HMBC data of compound **1** (400 MHz, 100 MHz; CD_3_OD).

Position	*δ_C_*	*δ_H_* (*J* in Hz)	COSY	HMBC
1	149.0	8.11 (s, 1H)		C-2,3,5,8,1′
2	126.2			
3	179.8			
4	119.3	6.86 (s, 1H)		C-2,5,7
5	155.8			
6				
7a	60.3	4.17 (d, *J* = 15.5, 1H)		C- 4,5
b		4.23 (d, *J* = 15.5, 1H)		
8	199.7			
9	31.4	2.68 (s, 3H)		C-2,8
1′	128.7			
2′	154.0			
3′	129.2	7.34 (dd, *J* = 7.9, 1.5, 1H)	H-4′	C-1′,2′,4′
4′	133.2	7.49–7.41 (m, 1H)	H-3′,5′	C-2′,3′
5′	121.5	7.10–7.01 (m, 1H)	H-4′,6′	C-4′,6′
6′	118.1	7.10–7.01 (m, 1H)	H-5′	C-1′,2′,4′,5′,5

**Table 2:** ^1^H, ^13^C NMR, and HMBC data of compound **2** (400 MHz, 100 MHz; CD_3_OD).

Position	*δ_C_*	*δ_H_* (*J* in Hz)	HMBC
2	78.5		
3a	43.0	1.87 (dd, *J* = 13.4, 9.8, 1H, Ha-3)	C-2,4,10,2′,3′
b		2.24 (dd, *J* = 13.4, 6.1, 1H, Hb-3)	
4	63.5	4.96–4.86 (m, 1H)	C-3,9,10
5	132.4	8.07 (s, 1H)	C-4,6,7,9,10,1′
6	131.5		
7	131.0	7.73 (dd, *J* = 8.5, 1.9, 1H)	C-6,9,1′
8	119.0	6.90 (d, *J* = 8.5, 1H)	C-6,7,9,10
9	160.6		
10	127.2		
1′	193.1	9.84 (s, 1H)	C-5,6,7
2′	29.7	1.48 (s, 3H)	C-2,3,3′
3′	26.1	1.36 (s, 3H)	C-2,3,2′

## Supplementary Materials

The following are available online. Figure S1: The picture of *Hericium* sp. WBSP8. Figure S2: Flow diagram of extraction and isolation procedure of compounds **1**–**9** as described in detail in the Materials and Methods. Figure S3: ^1^H NMR (400 MHz, CD_3_OD) spectrum of compound **1**. Figure S4: ^13^C NMR (400 MHz, CD_3_OD) spectrum of compound **1. Figure S5**: DEPT135 NMR (400 MHz, CD_3_OD) spectrum of compound **1**. Figure S6: DEPT90 NMR (400 MHz, CD_3_OD) spectrum of compound **1**. Figure S7: ^1^H-^13^C HSQC spectrum of compound **1** (400 MHz, CD_3_OD). Figure S8: ^1^H-^13^C HMBC spectrum of compound **1** (400 MHz, CD_3_OD). Figure S9: ^1^H-^1^H COSY spectrum of compound **1** (400 MHz, CD_3_OD). Figure S10: HRTOFMS spectrum of compound **1**. Figure S11: ^1^H NMR (400 MHz, CD_3_OD) spectrum of compound **2**. Figure S12: ^1^H NMR (400 MHz, CD_3_Cl_3_) spectrum of compound **2**. Figure S13: ^13^C NMR (400 MHz, CD_3_OD) spectrum of compound **2**. Figure S14: DEPT135 NMR (400 MHz, CD_3_OD) spectrum of compound **2**. Figure S15: DEPT90 NMR (400 MHz, CD_3_OD) spectrum of compound **2**. Figure S16: ^1^H-^13^C HMQC spectrum of compound **2** (400 MHz, CD_3_OD). Figure S17. ^1^H-^13^C HMBC spectrum of compound **2** (400 MHz, CD_3_OD). Figure S18. HRTOFMS spectrum of compound **2**. Table S1: Comparison of observed optical rotation of compound **2** with that of similar compounds. Table S2: MS, ^1^H, and ^13^C NMR data of known compounds.
