# Supplementary material for: Discovery of Antifungal and Biofilm Preventative Compounds from Mycelial Cultures of a Unique North American Hericium sp. Fungus
Source: Molecules. 2020 Feb 20;25(4):963. doi: 10.3390/molecules25040963 (PMC7070493; doi:10.3390/molecules25040963)
Supplement: Supplementary file 1 [file molecules-25-00963-s001.pdf]

Supplementary data

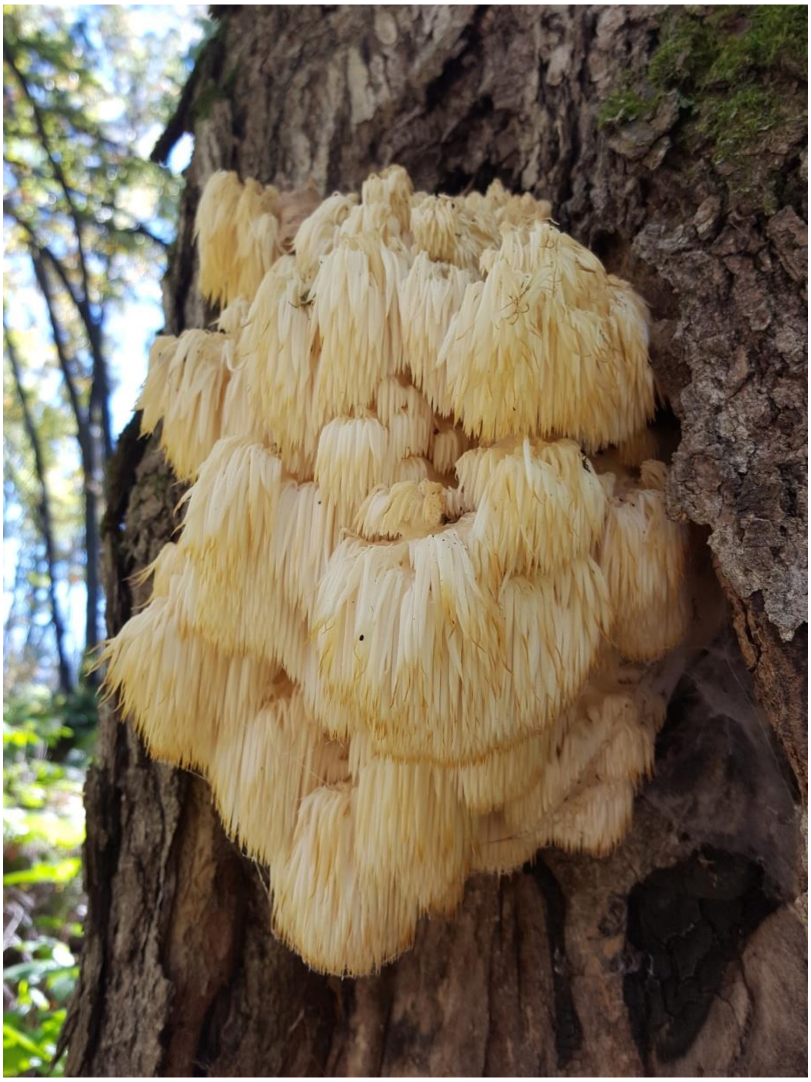

Figure S1. The picture of *Hericium* sp. WBSP8.

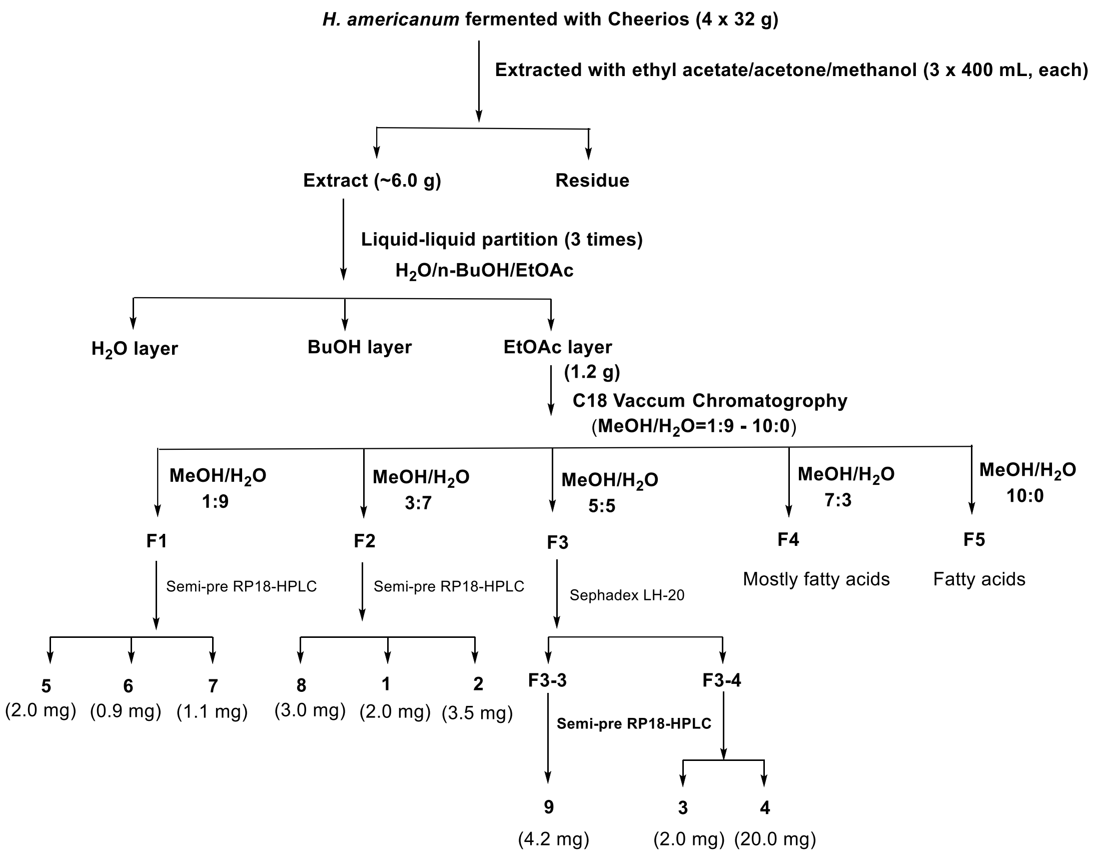

Figure S2. Flow diagram of extraction and isolation procedure of compounds 1-9 as described in detail in the Materials and Methods.

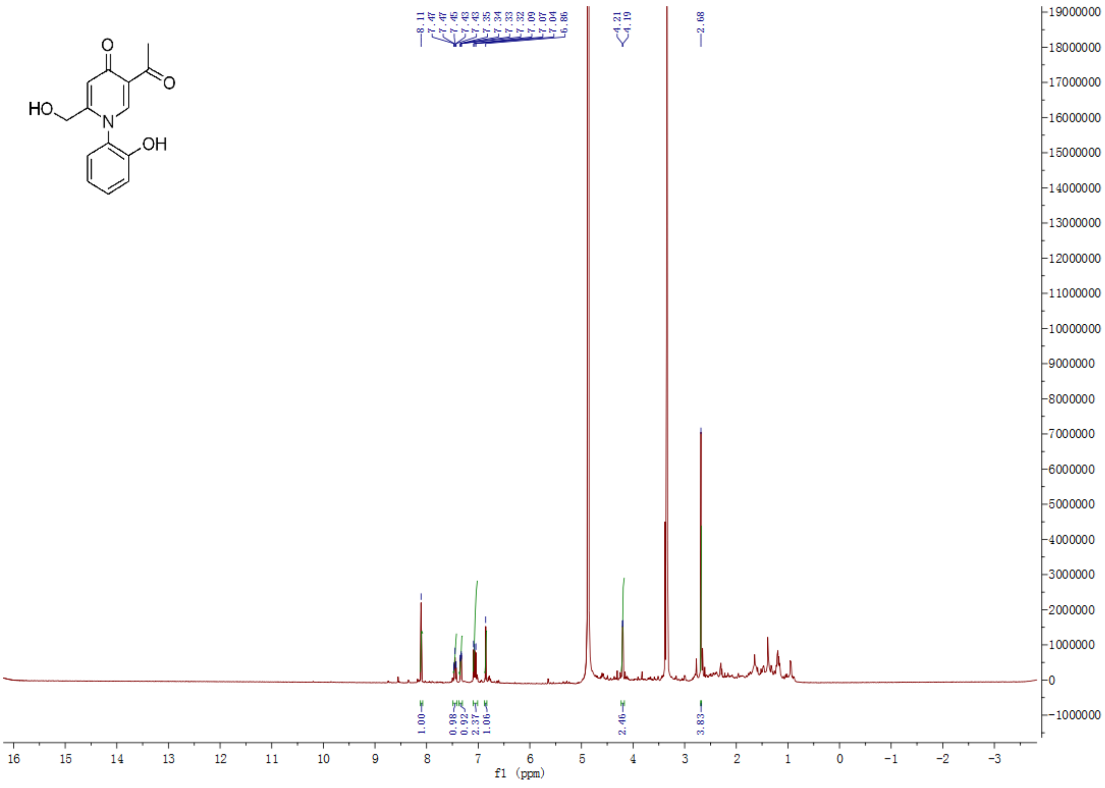

Figure S3. ^{1}H NMR (400 MHz, CD_3OD) spectrum of compound **1**

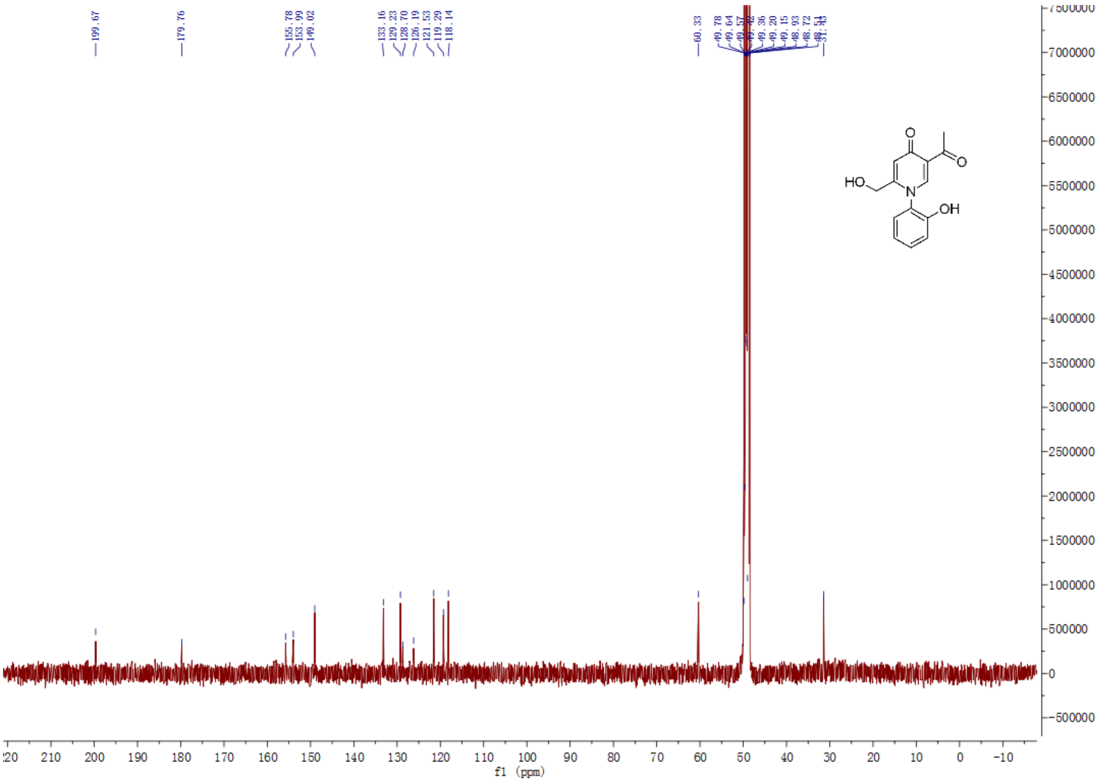

Figure S4. ^{13}C NMR (400 MHz, CD_3OD) spectrum of compound **1**

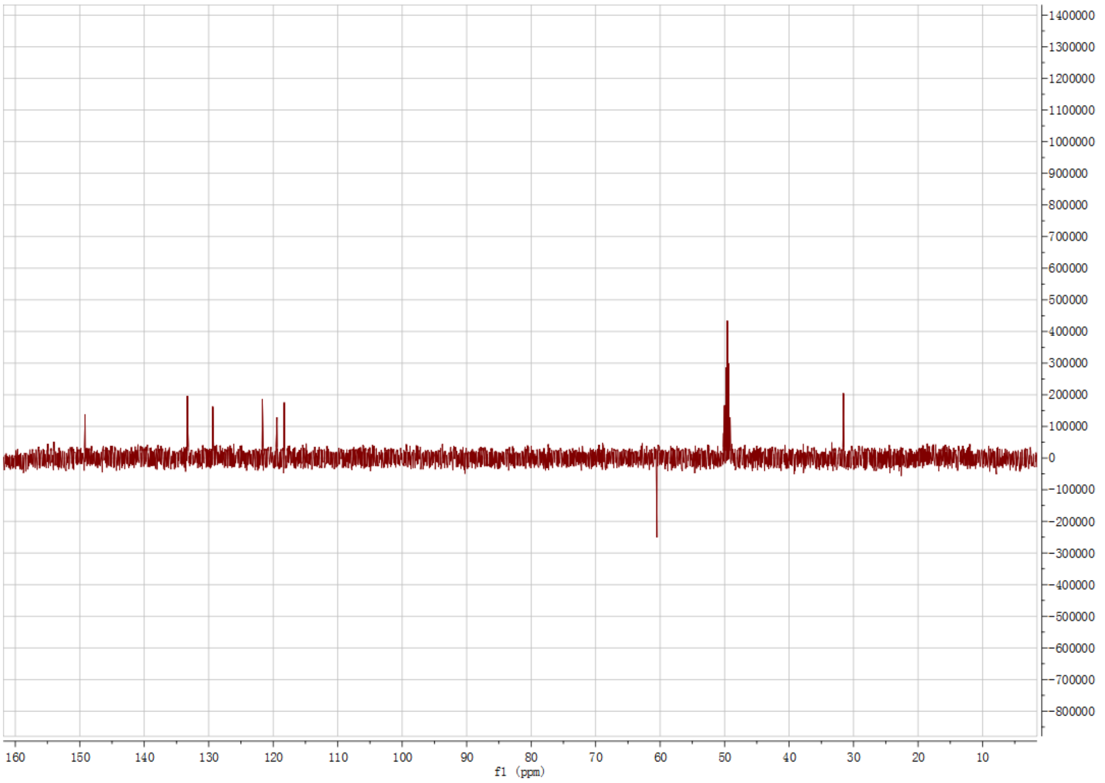

Figure S5. DEPT135 NMR (400 MHz, CD_3OD) spectrum of compound **1**

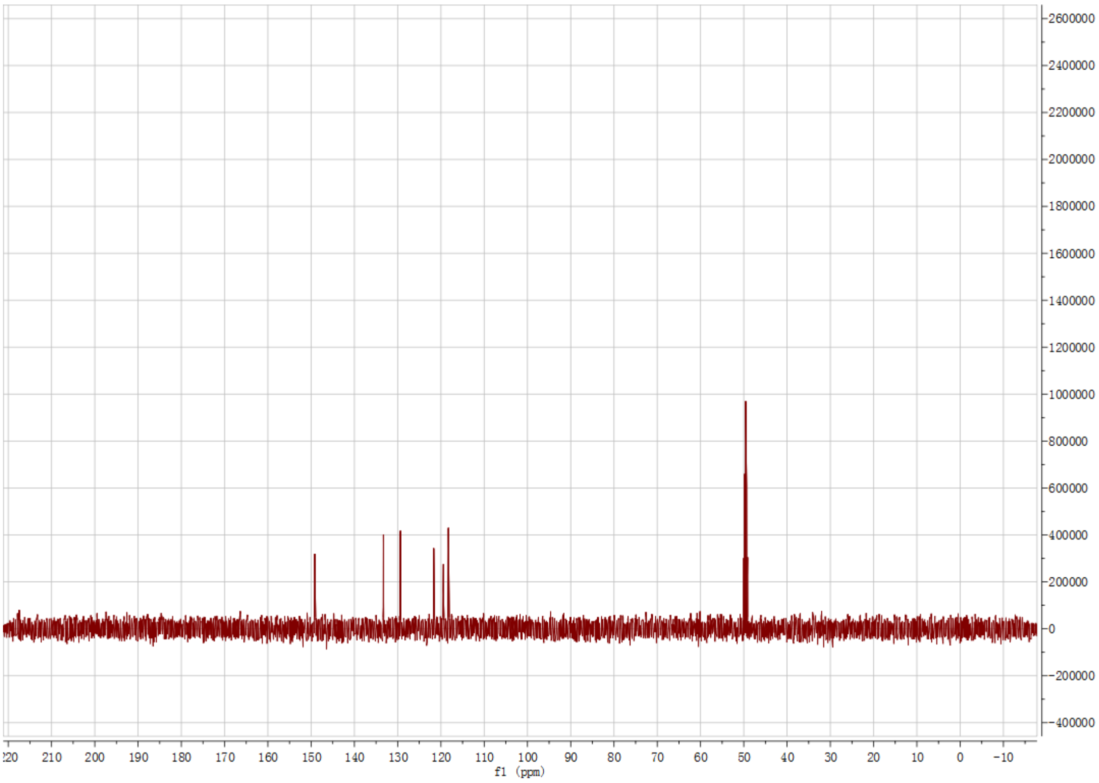

Figure S6. DEPT90 NMR (400 MHz, CD_3OD) spectrum of compound **1**

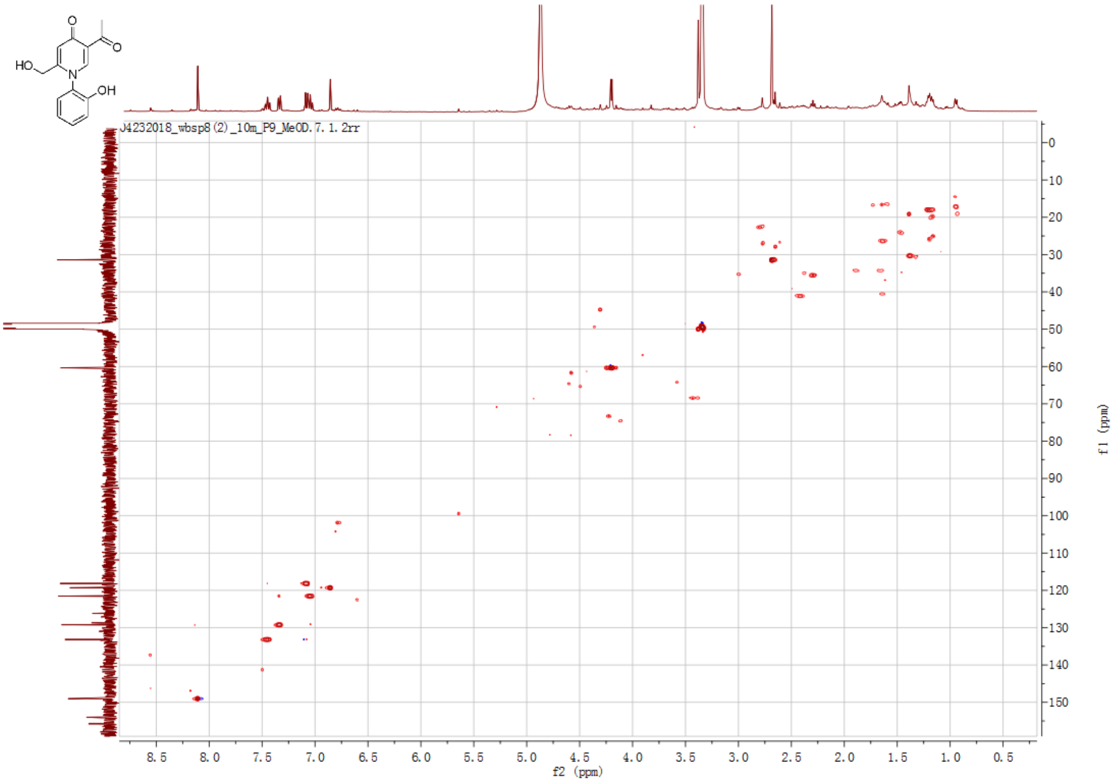

Figure S7. ^{1}H-^{13}C HSQC spectrum of compound **1** (400 MHz, CD_3OD)

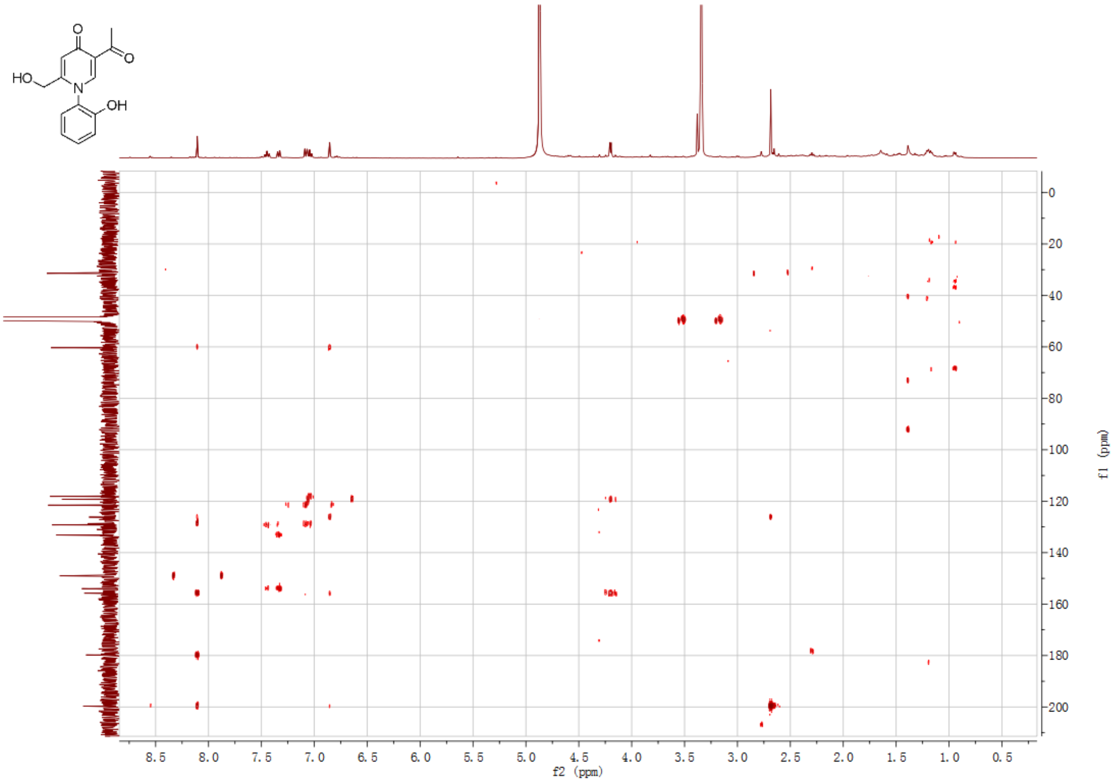

Figure S8. ^{1}H-^{13}C HMBC spectrum of compound **1** (400 MHz, CD_3OD)

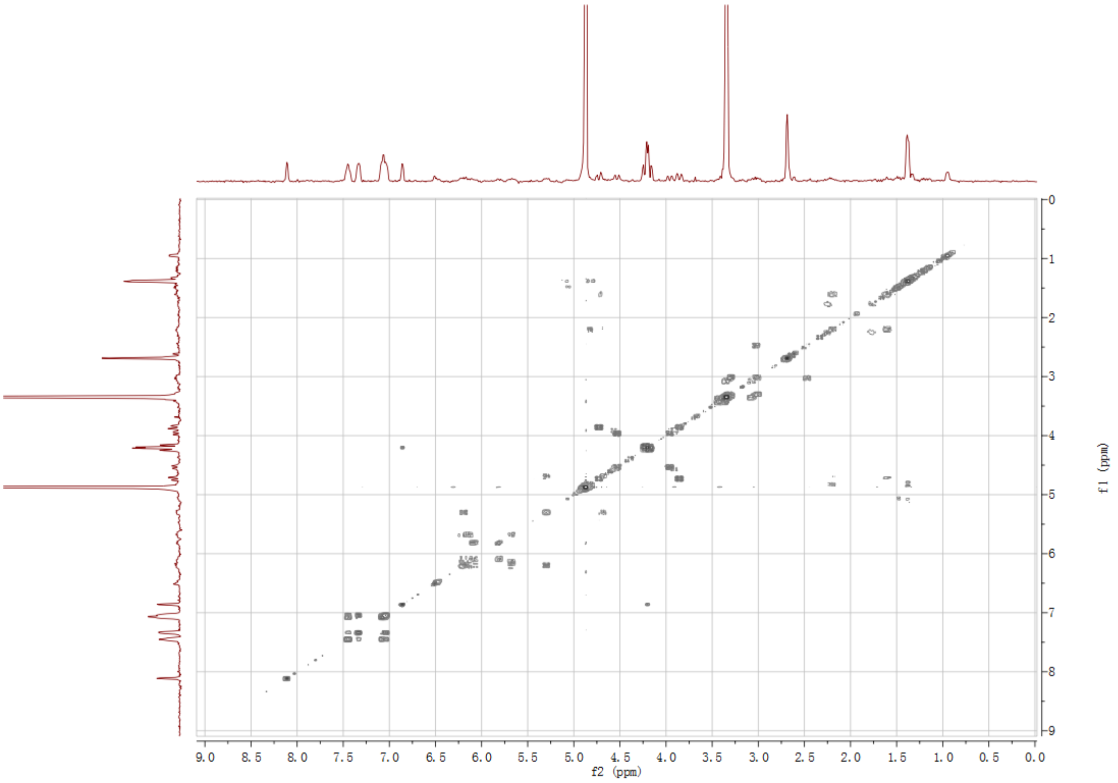

Figure S9. ^{1}H-^{1}H COSY spectrum of compound **1** (400 MHz, CD_3OD)

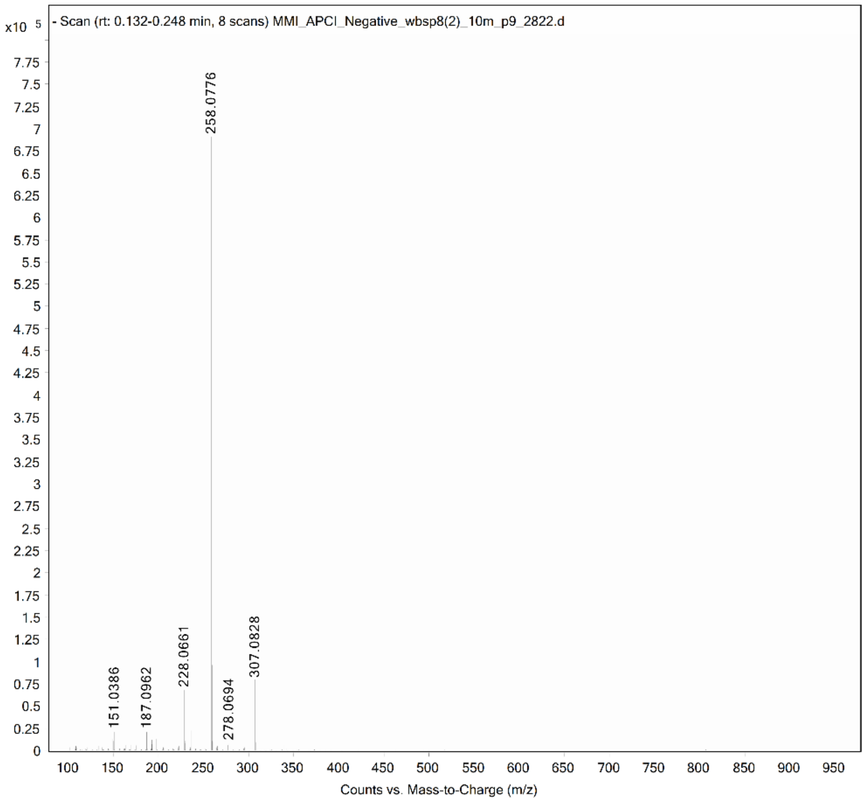

Figure S10. HRTOFMS spectrum of compound **1**

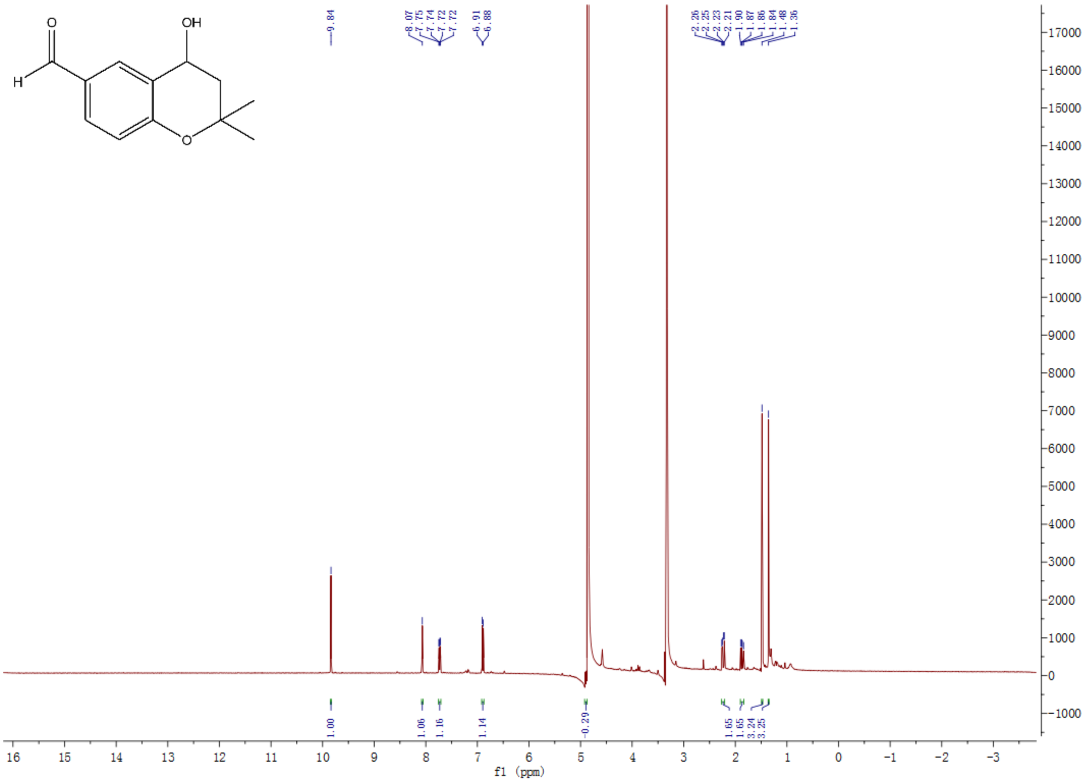

Figure S11. ^{1}H NMR (400 MHz, CD_3OD) spectrum of compound **2**

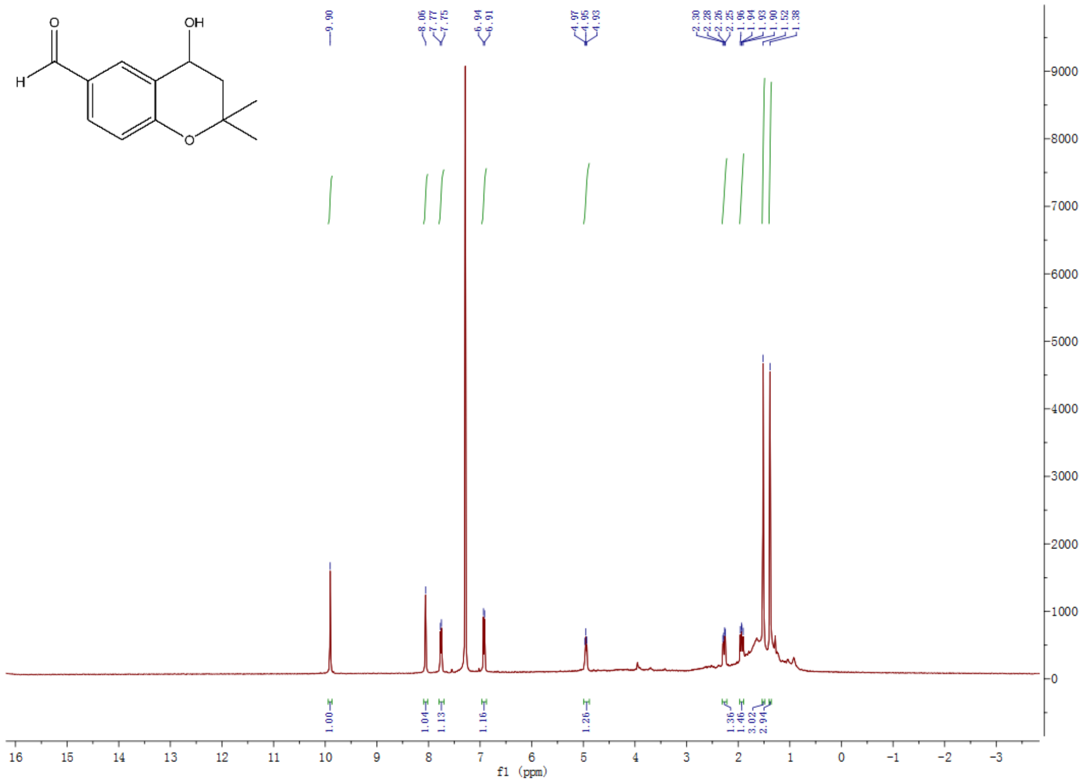

Figure S12. ^{1}H NMR (400 MHz, CD_3Cl_3) spectrum of compound **2**

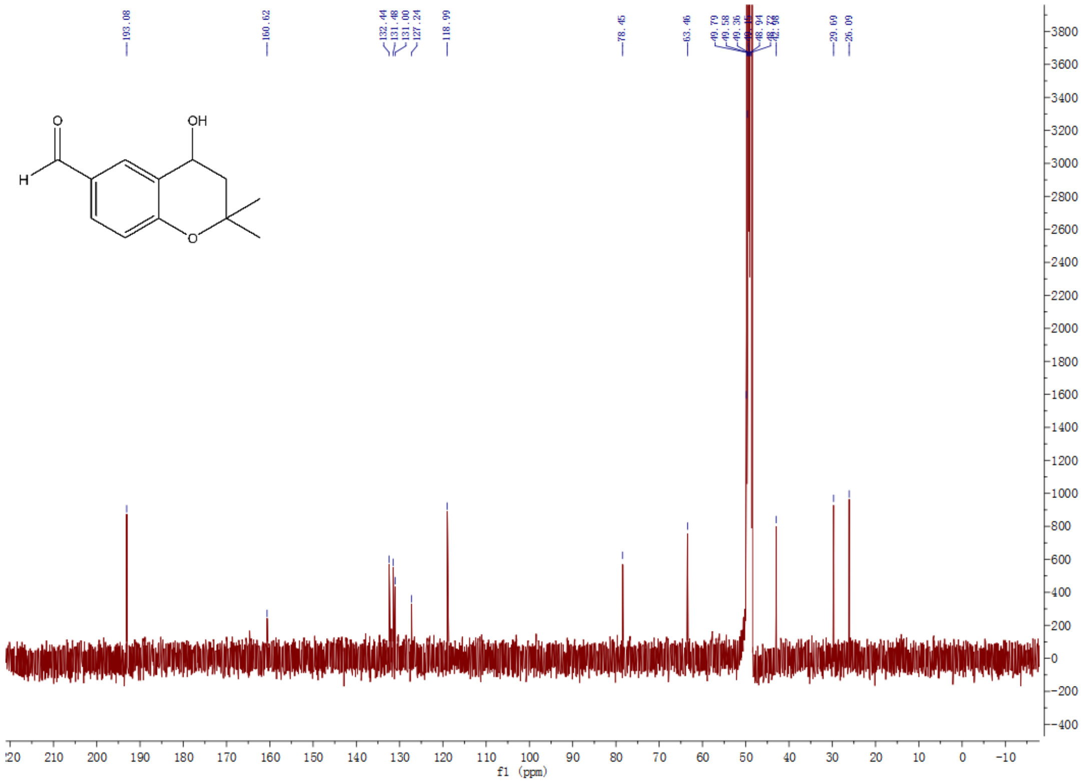

Figure S13. ^{13}C NMR (400 MHz, CD_3OD) spectrum of compound **2**

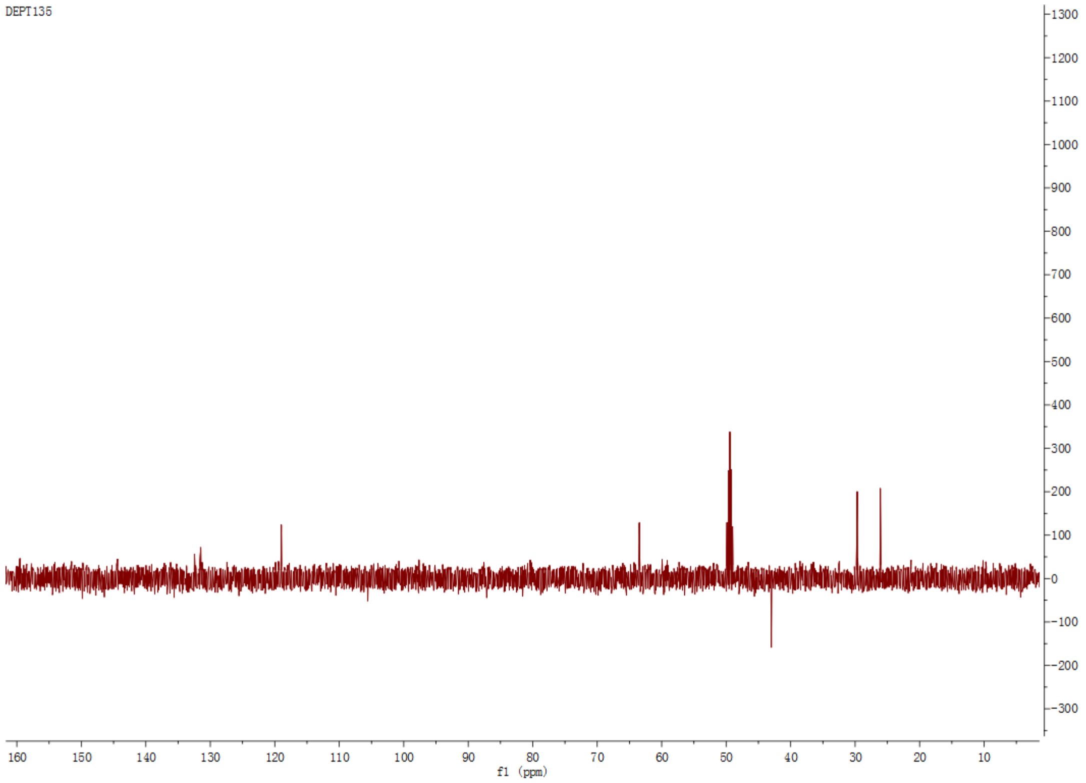

Figure S14. DEPT135 NMR (400 MHz, CD_3OD) spectrum of compound **2**

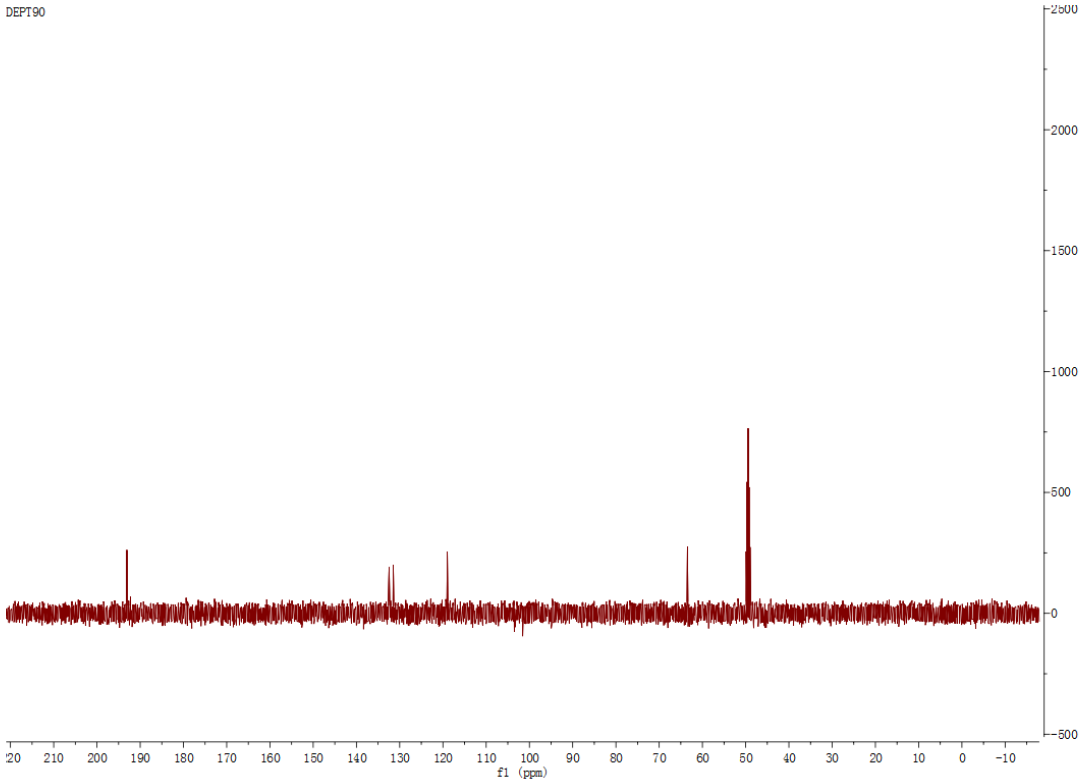

Figure S15. DEPT90 NMR (400 MHz, CD_3OD) spectrum of compound **2**

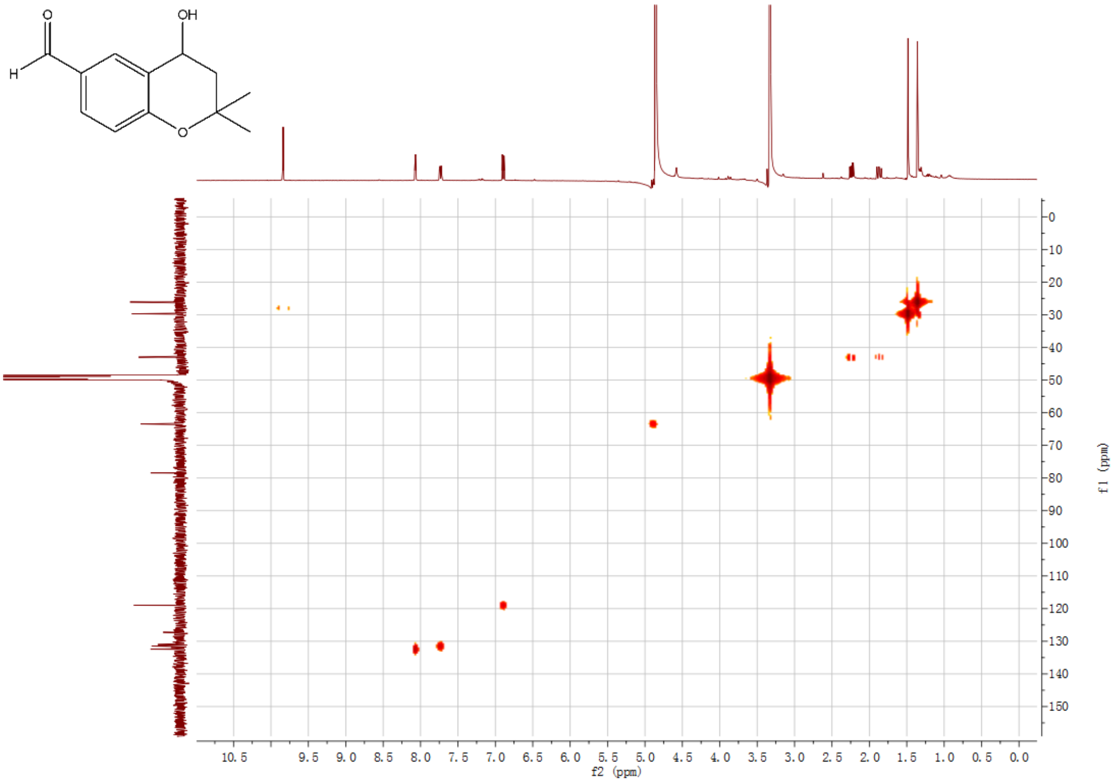

Figure S16. ¹H-¹³C HMQC spectrum of compound **2** (400 MHz, CD3OD)

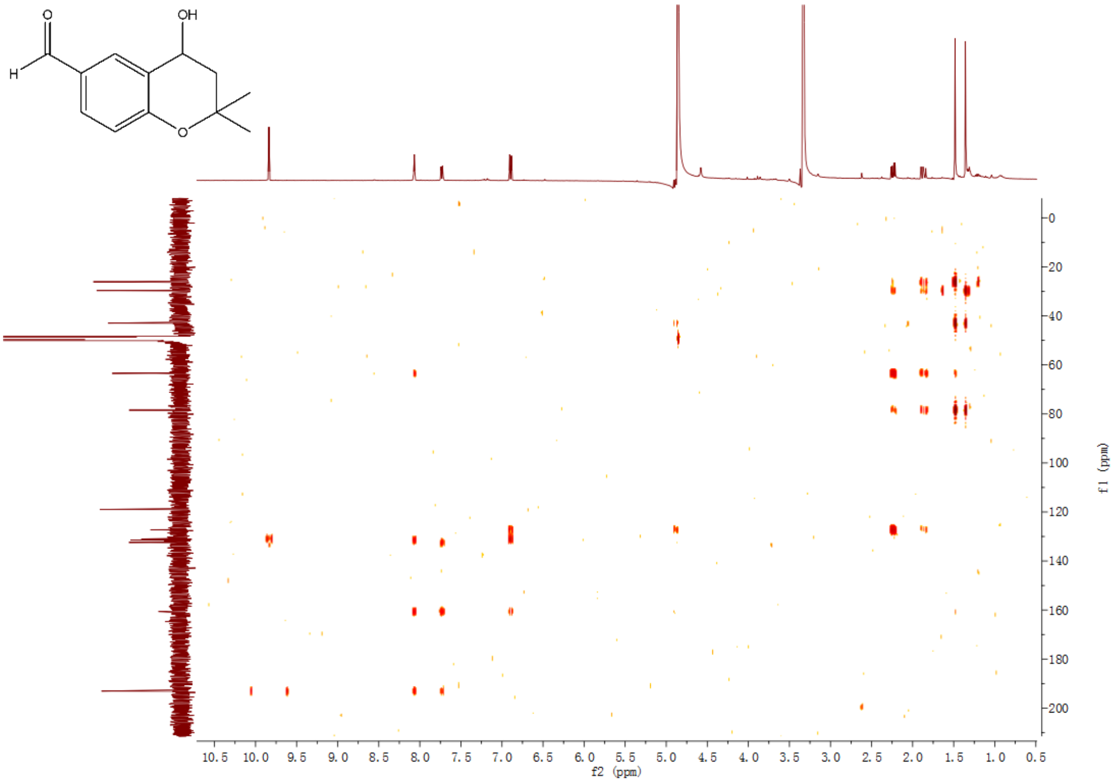

Figure S17. ¹H-¹³C HMBC spectrum of compound **2** (400 MHz, CD_3OD)

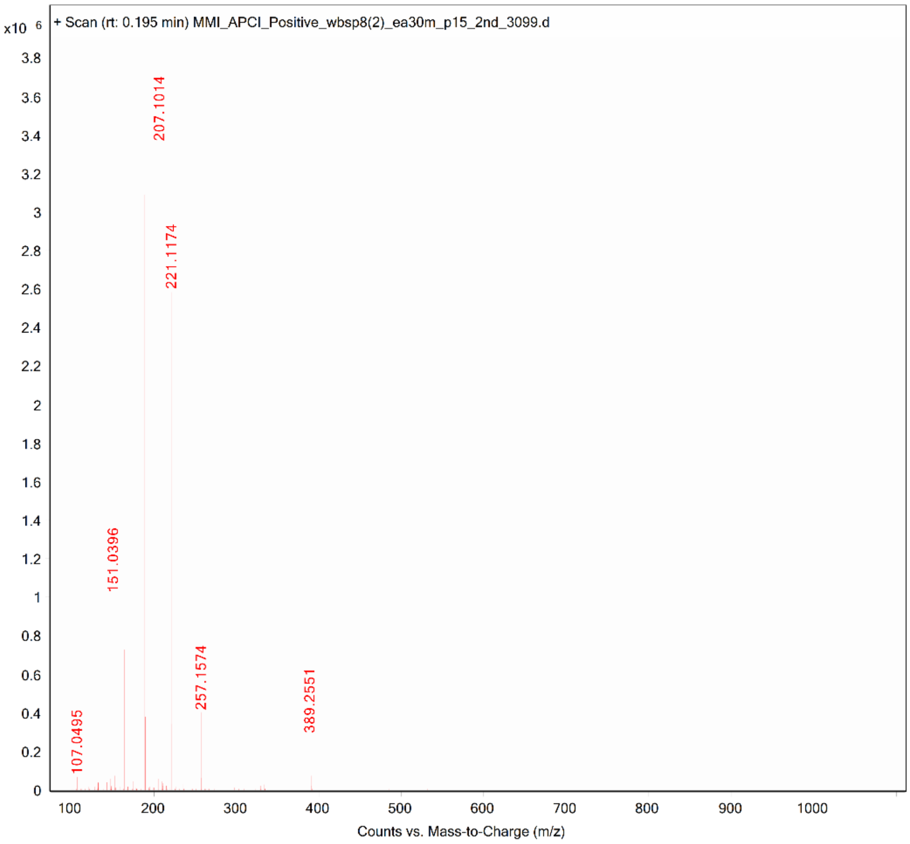

Figure S18. HRTOFMS spectrum of compound **2**

Table S1. Optical rotation data of compound **2** and similar compounds

Compound	Structure	Solvent	$[\alpha]_D^{25}$ (degree)
(*S*)-1-(4-hydroxy-2,2-dimethylchroman-6-yl)ethan-1-one [1]	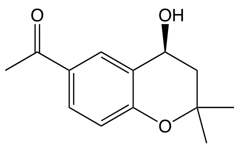	c = 0.26, MeOH	+68.6
(*R*)-1-(4-hydroxy-2,2-dimethylchroman-6-yl)ethan-1-one [1]	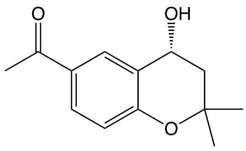	c = 0.25, MeOH	-79.8
Compound **2**	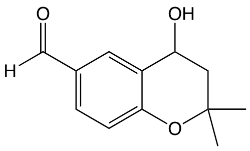	c = 0.12, MeOH	+10.0

[1] Sun *et.al.*,Chromane Enantiomers from the Flower Buds of *Tussilago farfara* L. and Assignments of Their Absolute Configurations. *Chem. Biodiversity* 2019, 16, e1800581

Table S2. MS, ^{1}H and ^{13}C NMR Data of Known Compounds

Compounds	MS and NMR data
4-chloro-3,5-dimethoxy benzaldehyde (**3**)	HR-MS ($APCI^+$): m/z 218.0657 $[M+NH_4]^+$. ^{1}H NMR (400 MHz, $CDCl_3$) δ 9.93 (s, 1H), 7.12 (s, 2H), 3.99 (s, 6H). ^{13}C NMR (101 MHz, $CDCl_3$) δ 191.32, 157.13, 135.53, 117.71, 105.68, 56.98.
2-chloro-1,3-dimethoxy-5-methylbenzene (**4**)	HR-MS ($APCI^+$): m/z 187.0516 $[M+H]^+$. ^{1}H NMR (400 MHz, $CDCl_3$) δ 6.40 (s, 2H), 3.86 (s, 6H), 2.32 (s, 3H). ^{13}C NMR (101 MHz, $CDCl_3$) δ 156.03, 137.80, 107.76, 105.87, 56.50, 22.24.
(4-chloro-3,5-dimethoxy phenyl) methanol (**5**)	HR-MS ($APCI^-$): m/z 201.0353 $[M-H]^-$. ^{1}H NMR (400 MHz, $CDCl_3$) δ 6.63 (s, 2H), 4.69 (s, 2H), 3.91 (s, 6H).13C NMR (101 MHz, CDCl3) δ 156.57, 140.84, 109.98, 103.37, 65.59, 56.74.
3,6-bis(hydroxymethyl) -2-methyl-4H-pyran-4-one (**6**)	HR-MS ($APCI^-$): m/z 215.0118 $[M-H]^-$. ^{1}H NMR (400 MHz, CD_3OD) δ 7.46 (s, 2H), 3.97 (s, 6H). ^{13}C NMR (101 MHz, CD_3OD) δ 171.18, 157.69, 130.98, 111.55, 106.96, 57.15.
4-chloro-3,5-dimethoxy benzoic acid (**7**)	HR-MS ($APCI^+$): m/z 171.0651 $[M+H]^+$. ^{1}H NMR (400 MHz, CD_3OD) δ 6.42 (s, 1H), 4.52 (s, 2H), 4.43 (s, 2H), 2.47 (s, 3H). ^{13}C NMR (101 MHz, CD_3OD) δ 181.53, 170.71, 167.96, 124.87, 111.76, 61.24, 55.11, 17.57.
5-hydroxy-6-(1-hydroxy ethyl)isobenzofuran-1 (3H)-one (**8**)	HR-MS ($APCI^+$): m/z 195.0651 $[M+H]^+$. ^{1}H NMR (400 MHz, CD_3OD) δ 7.89 (s, 1H), 6.93 (s, 1H), 5.28 (s, 2H), 5.18 (q, *J* = 6.4 Hz, 1H), 1.46 (d, *J* = 6.4 Hz, 3H). ^{13}C NMR (101 MHz, CD_3OD) δ 174.10, 161.98, 150.07, 136.79, 124.05, 117.34, 108.86, 70.83, 66.19, 24.13.
Erinacines E (**9**)	$[\alpha]^{18}D$: -144 (c 0.50, MeOH). HR-MS ($APCI^-$): m/z 431.2439 $[M-H]^-$. ^{1}H NMR (400 MHz, CD_3OD) δ5.63(m,1H), 4.96 (s,1H), 4.72(br.s, 1H), 4.23(d, *J*= 6.23, 1H), 3.93 (d,J=12.28, 1H), 3.92(s, 1H), 3.24(d, J=12.28,1H), 3.13(m, 1H),2.96(qq, *J*=6.60, 6.96,1H), 2.88(br.d, J=12.46, 1H), 2.68(m, 1H), 2.62(m, 1H) 2.32(m, 2H), 1.70(m, 1H), 1.55(m, 3H), 1.31(m, 1H), 1.11(s, 3H), 1.02(d, *J*=6.96, 3H), 1.00(d, *J*=6.96, 3H), 0.98(s, 3H). ^{13}C NMR (101 MHz, CD_3OD) δ 142.74, 140.34, 138.89, 124.19, 106.65, 97.65, 80.36, 76.05, 75.08, 72.38, 66.74, 51.0,44.44, 44.14, 42.43, 40.29, 38.32, 32.11, 29.38, 28.69, 28.32, 25.20, 22.26, 17.54.
